# The effect of circuit resistance training, empagliflozin or “vegeterranean diet” on physical and metabolic function in older subjects with type 2 diabetes: a study protocol for a randomized control trial (CEV-65 trial)

**DOI:** 10.1186/s12877-019-1219-7

**Published:** 2019-08-22

**Authors:** Assaf Buch, Roy Eldor, Ofer Kis, Lital Keinan-Boker, Ayelet Dunsky, Amir Rubin, Adar Lopez, Yael Sofer, Etty Osher, Yonit Marcus, Naftali Stern

**Affiliations:** 1 Institute of Endocrinology, Metabolism and Hypertension, Tel Aviv Sourasky Medical Center, Tel-Aviv, Israel; 2 Tel Aviv Sourasky Medical Center, The Sagol Center for Epigenetics of Aging and Metabolism, the Institute of Endocrinology, Metabolism and Hypertension, Tel-Aviv, Israel; 3 The Sackler Faculty of Medicine Tel-Aviv University, Tel-Aviv, Israel; 4 The Faculty of Health Sciences, Ariel University, Ariel, Israel; 5 School of Public Health, University of Haifa, Haifa, Israel; 6 Israel Center for Disease Control, Israel Ministry of Health, Ramat Gan, Israel; 7 The Academic College at Wingate, Wingate Institute, Netanya, Israel

**Keywords:** Weight loss, Diabetes, Frailty, Muscle function, Muscle mass, Sarcopenia

## Abstract

**Background:**

Treatment of the older diabetic individual comprises a therapeutic challenge. Currently little scientific evidence exists depicting the best approach to type 2 diabetes treatment in this growing sub-population of patients. The purpose of this study is to assess the effects of a modified plant-based Mediterranean diet (“vegeterranean” diet), circuit resistance training (CRT) and empagliflozin, separately or in combination, on body composition and physical function in older subjects with type 2 diabetes. The rationale for this study is to assess three interventions associated with a negative energy/caloric balance (increased caloric use in exercise, caloric restriction in the “vegeterranean” diet and caloric wasting by glycosuria with empagliflozin), their interaction and effect on body composition and physical function.

**Methods:**

One hundred and twenty men and women ≥65 years of age with type 2 diabetes, and low levels of physical activity will be randomized (1:1:1 manner, gender stratified) for 10 weeks to one of 3 parallel arms: CRT consisting of 3 home sessions/week; ad-libitum plant-based Mediterranean diet (limited consumption of eggs, dairy and fish, avoidance of red meat and poultry) or empagliflozin 10 mg/day. After 10 weeks CRT will be added to the empagliflozin and diet arms for an additional 10 weeks. Allocation concealment and blinding of primary outcome assessors will be implemented. Efficacy will be determined by assessment of lean body mass, body weight, frailty and functional status, sarcopenia, HbA1c and quality of life questionnaires. Safety will be evaluated by routine monitoring of adverse events. This study was approved by the Tel-Aviv Sourasky Medical Center Institutional Review Board.

**Discussion:**

The combination and comparison of these diverse interventions to metabolic control may lead to better understanding of their mechanism of action with potential clinical implications in older individuals. Also, this study will provide evidence of the effectiveness of these interventions on delaying the progression from diabetes to sarcopenia and/or frailty.

**Trial registration:**

ClinicalTrials.gov PRS: NCT03560375. Last registration date (last update): 06/06/2018. The trial was a-priori registered before actual recruitment of subjects.

**Electronic supplementary material:**

The online version of this article (10.1186/s12877-019-1219-7) contains supplementary material, which is available to authorized users.

## Background

Longevity has been steadily increasing over the past several decades. Life expectancy in 65-year old Israeli adults is ~ 20 years, leaving an ever expanding time window for the evolution of loss of health and function [1]. In western countries, the proportion of people over age 60 is increasing faster than any other group [2]. Diabetes rates (mainly type 2 diabetes mellitus -T2DM) are increasing in general and in older adults [4]. In the past the older population was characterized by low body weight and malnutrition in contrast to the present in which rates of obesity, metabolic syndrome and T2DM are increasing [3, 4]. Due to its chronic nature, T2DM presents a challenge to both patients and healthcare systems in maintaining adherence to treatment and accompanying lifestyle changes [5, 6].The risk for T2DM related complications is age related and is accompanied by functional limitation and comorbidities [7, 8]. One morbidity associated with diabetes in the older population is sarcopenia [9] – a phenomenon of age-related loss of skeletal muscle mass, strength and function. The decreased muscle mass is associated with loss of strength, increased likelihood of falls, and loss of autonomy [10].

Whereas weight reduction and physical activity are cornerstones of T2DM treatment and are likewise recommended as first-line treatment in adults [8], the older subpopulation is underrepresented in clinical trials [4]. Numerous diet regimens have been shown to improve T2DM control in relatively short clinical trials including the Mediterranean diet (Med-diet) [11], vegetarian / vegan [12, 13] and low-carb diets [12, 14, 15]. However, in older individuals, low-carb diets resulted in contradicting results [14, 15]; a meta-analysis of 10 randomized controlled trials (RCTs) comprising 1376 participants (age range 54–63 yrs.) showed that in the first year of intervention (3 or 6 months), a low carbohydrate diet (< 45% of total caloric consumption) was followed by a 0.34% lower HbA1c compared with a high carbohydrate diet (45–60% of total caloric consumption). However, at 1 year or later, HbA1c was similar in the two groups with higher dropout rates in the low carbohydrate diet groups [14]. Med-diet with a vegetarian pattern (the “vegeterranean” diet- V-Med diet) has a higher portion of carbohydrates when compared to low-carb diets, accompanied with high levels of fat (mainly unsaturated). To the best of our knowledge the V-Med diet has never been studied in older patients with T2DM.

In addition to diet, physical activity (PA) is an integral part of T2DM therapy, as it is associated with a lower risk of cardiovascular disease and mortality [16]. However, adherence to exercise in older adults is low and studies in older adults performing unsupervised physical activity are scarce and have shown limited improvement in performance when compared to fully supervised programs [17, 18]. The cost of supervised training programs limits their widespread use and results in a need to develop more achievable evidence-based “shorter duration” home-training regimens [17] in older people with T2DM.

In addition to low adherence to PA, insulin resistance, a key pathogenic process in T2DM and a common condition in older individuals, is associated with “anabolic resistance”, which interferes with any attempted increase or preservation of skeletal muscle mass [19]. Anabolic resistance is associated with sarcopenic obesity and frailty [20] and requires careful management of weight reduction while performing PA [21].

Recently, we have shown that circuit resistance training (CRT) is effective in increasing muscle mass and especially muscular strength in older adults with diverse health conditions [22]. CRT performed with minimal supervision (performed in the patient’s home) was never studied in a Randomized Controlled Trial (RCT) in healthy or diabetic older individuals.

Empagliflozin is a new antihyperglycemic medication that induces glycosuria via inhibition of the renal sodium-glucose transporter-2 (SGLT-2) [23–25]. Independent of its anti-hyperglycemic effect, empagliflozin is cardio-protective in diabetic patients with pre-existing cardiovascular disease [26, 27]. Treatment with empagliflozin and other SGLT-2 inhibitors results in weight loss (through urinary caloric loss) and potential compensatory hyperphagia without a significant change in resting metabolic rate (RMR) [24, 25, 28, 29].

In this study we aim to compare the effect of a V-Med diet, CRT and empagliflozin separately or in combination on metabolic and anthropometric parameters in older subjects with T2DM. Both SGLT2 inhibitors and exercise lead to increased caloric expenditure and compensatory hyperphagia; SGLT2 inhibitors and diet lead to weight loss and all three interventions reduce insulin resistance and lower circulating insulin levels [30, 31]. Therefore, the combination of and comparison among these diverse approaches to metabolic control may lead to better understanding of their mechanism of action with potential clinical implications in older individuals.

In conclusion, treatment of the older diabetic individual comprises a therapeutic challenge. Currently little scientific evidence exists depicting the best approach to T2DM treatment in this growing sub-population of patients. In this study we aim to better quantify the short-term effects of diet, physical activity and drug therapy, alone or in combination, on metabolic and anthropometric parameters as well as on physical function in older individuals with T2DM.

### Objectives

The aim of the study is to assess the effects of different medical treatment modalities for T2DM, separately and combined in community dwelling older subjects, on metabolic, functional and anthropometric parameters.

#### Primary aims and hypotheses

##### Aim

To evaluate the short-term efficacy (10–20 weeks) of CRT, a V-Med diet separately or in combination with CRT, or empagliflozin separately or in combination with CRT, on the body weight, body composition and functional status in community dwelling older subjects with T2DM.

##### Hypothesis 1

We expect that the increase in lean body mass (LBM) will be superior in the CRT arm vs. the other groups after 10 weeks. After 20 weeks the relative increase in LBM will be higher in the V-Med diet + CRT compared to the empagliflozin + CRT group.

##### Hypothesis 2

We expect that the functional status would improve the most in the CRT arm vs. the other groups after 10 weeks. After 20 weeks, functional improvement will be larger in the combined therapy of V-Med diet + CRT as compared to the empagliflozin + CRT combination.

#### Specific secondary aims

1. To profile community dwelling older people with T2DM in terms of overall health status, body composition, physical and metabolic function.
2. To evaluate the effects of the three study arms on glycemic control, insulin resistance, hormonal and lipid profiles, blood pressure and energy expenditure in the community dwelling older subjects with T2DM.
3. To assess the effects of the three study arms on sarcopenia and frailty measures in the community dwelling older subjects with T2DM.
4. To evaluate whether or not there is a synergistic effect of either medical treatment with empagliflozin or the V-Med diet with the CRT intervention on weight reduction and functional status in community dwelling older subjects with T2DM. This will be done by comparing among each of the interventions alone, and with its combined effect with the other listed co-interventions.

## Methods/design

### Trial design

**Fig. 1:**
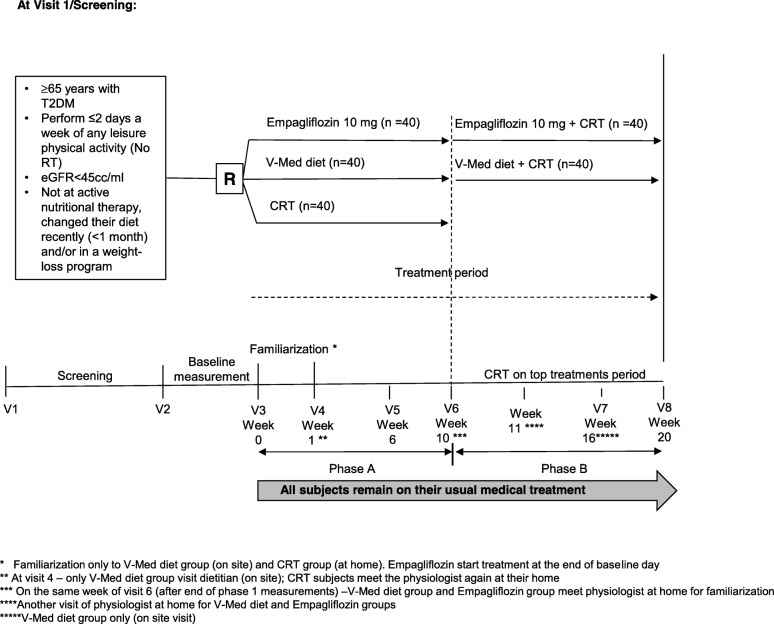
Study flow. Abbreviations: CRT, circuit resistance training; R, randomization; SGLT2, Sodium glucose transport 2; T2DM, type 2 diabetes mellitus; V, visit (on site); V-Med diet, vegeterranean diet

This is a single center, open label, parallel group, exploratory clinical trial of CRT for 10 weeks; V-Med diet for 10 weeks and then CRT on top of diet for 10 weeks; empagliflozin 10 mg/day for 10 weeks and then CRT on top of drug therapy for 10 weeks (Fig. 1) in older subjects with T2DM. This trial will be conducted in conformance with Good Clinical Practices (GCP). The duration of the trial will be up to 25 weeks (with up to 8 clinic visits) for each subject. This will include a 1-week screening period (Visit 1 to Visit 2); a 10-week single treatment period (Visit 2 or 3 to Visit 6); a 10-week combination treatment period. Approximately 120 subjects ≥65 years of age with T2DM, diagnosed in accordance with American Diabetes Association guidelines and who meet all enrollment criteria will be randomized. The study will be conducted between May 2018 to September 2020. Information on the trial is disclosed at clinicaltrail.gov (trial no. NCT03560375). The trial will be disclosed according to the requirements of the International Committee of Medical Journal Editors (ICMJE) [32]. This study was approved by the Tel-Aviv Sourasky Medical Center Institutional Review Board. Any important protocol modifications will be reported for approval of the Institutional Review Board.

### Study setting

The study will take place at the institute of Endocrinology, Metabolism, and Hypertension (IEMH), Tel-Aviv Sourasky Medical Center (TASMC). We will use a non-probability sampling method composed of patients aged 65 years and older with T2DM visiting the various out-patient clinics in the IEMH (e.g., endocrinology, osteoporosis, diabetes, metabolic syndrome clinics). Further recruitment, if necessary, will be based on retired employees from Tel Aviv municipality and from physician clinics in health maintenance organizations (HMO’s) located in Tel Aviv and its area. Potential participants from all recruitment sources will be shortly informed of the study and will be sent detailed information via e-mail or mail. Their eligibility will be assessed by the study physician (RE) and if willing to participate they will sign an informed consent.

### Eligibility

#### Major inclusion criteria

Subjects who have T2DM in accordance with American Diabetes Association guidelines [33] and:

1. Are ≥65 years of age (inclusive) on the day of signing the informed consent form (ICF). Subjects may be treated with any anti hyperglycemic agent other than an SGLT-2 inhibitor.
2. Perform ≤2 days a week of any leisure aerobic PA, who are able to walk independently either with or without an assistance device (cane or walker).
3. HbA1C ≥6.5% to ≤8%.

#### Major exclusion criteria

1. Recent use of steroid agents (< 6 months, replacement therapy is allowed)
2. Uncorrected hypothyroidism [thyroid stimulating hormone (TSH) > 6 mlU/L]
3. Diagnosis of malignancy within the past 5 years except for non-melanoma skin cancer
4. Severe kidney disease (eGFR< 45 cc/ml)
5. Active depression
6. Recent (≤6 months) or unstable cardiovascular condition; New York Heart Association (NYHA) Class 3 or higher congestive heart failure
7. Subjects with PA limiting pain due to advanced neuropathy
8. Subjects performing resistance training
9. Subjects who are in active nutritional therapy, changed their diet recently (< 1 month) and/or in a weight-loss program (actively losing weight)
10. Has other severe acute or chronic medical or psychiatric condition or laboratory abnormality that may increase the risk associated with study participation or may interfere with the interpretation of study results.

### Visit 1- screening

All potential eligible subjects will be invited for a screening Visit 1, during which the following information would be obtained:

- Informed consent.
- Physician interview and physical examination.
- A questionnaire adapted from the national Israeli health and nutrition survey (MABAT) for the older people which includes demographic details, health status, lifestyle as well as descriptive details addressing the functional and physical state.
- A meeting with the research assistance (AL) (blinded during all study) to assess adherence to the Med-diet, using the PrediMed screener which was recently adopted to Israel.
- A meeting with the study’s physiologist (OK) comprised of a short interview and frailty and functional assessments.

Screening information from all subjects will be documented and kept in a screening log. Comparisons of baseline characteristics of the included vs. the excluded subjects will be performed at the end of the trial (age, gender, etc.).

### Visit 2- day 1

Subjects that were confirmed as eligible at the end of Visit 1 will be considered as “included subjects”. During Visit 2, subjects will be assigned a 5-digit number (their 5 first ID number’s digits) and the following information will be collected:

- Following overnight fasting (12 h) patients will undergo: a) resting metabolic rate (RMR); b) Anthropometric assessment (then food is allowed); c) Fasting blood draw for routine glucose and hormonal profile; d) Blood Pressure (BP) (diastolic and systolic) and pulse measurements; e) eye examination.

#### Assignment of intervention

##### Allocation

At the end of Visit 2- subjects will be randomized at a ratio of 1:1:1 to each study group and stratified based on gender. Randomization will be conducted by a randomization software (computer generated random numbers) [34]. A third party who is not aware of research protocol will allocate patients into the study groups a priori to first recruitment using the randomization software and will create and keep the allocation list. Once recruitment for a particular baseline day and all baseline measurements are finished, a mediator in the IEMH (secretary) will contact the third party to reveal the allocations of the participants recruited and examined so far (allocation concealment).

##### Blinding

As mentioned we will use allocation concealment, such that at baseline all outcome assessors will be blinded to allocation. At follow up outcome assessors that will be blinded to the participants allocation will include: laboratory technicians (performing blood analysis); research assistant (AL) (in charge of nutritional analysis and the Med-diet questionnaire); ophthalmologist (for eye examination); endocrinologist (YM) (analyzing the 24–hour ambulatory blood pressure monitoring [ABPM]); another researcher (AD) (analyzing the quality of life questionnaires). The study physician (RE), nutritionist (AB), physiologist (OK) and physical instructor (AR) will not be blinded due to their role of instructing the patients on the intervention as well as collecting the following outcomes: body composition (automatic analysis but measured by AR); food intake and RMR (AB) and physical performance tests (OK).

### Intervention

#### Circuit resistance training (CRT)

Traditionally, the recommendations for physical activity in older adults (given by the ACSM and AHA) include 150 min of moderate intensity aerobic exercise/week and at least two resistance training exercise sessions (~ 60–90 min/session) /week [35]. As resistance training is considered to be the most appropriate form of intervention to decelerate muscle mass and strength loss [35] and keeping in mind the low adherence to the PA recommendations [17] we chose to focus on resistance exercise in the form of CRT. In this study subjects will be allocated to CRT which consists of 10 resistance exercises for different body parts (=1 circuit) repeated 12–15 times, using modest weights (approximately 40–60% of one repetition maximum, RM). Each exercise is expected to be completed within 30–40 s. The participant will move quickly (within 15–30 s) from one exercise to the next. The circuit will be repeated up to three times depending on the week of intervention. Thus, it will take about 30 min to complete an exercise session [22, 36]. Participants are expected to perform three full sessions per week on non-consecutive days. A summary of articles on CRT in older adults compiled in our meta-analysis are provided in ref. [22]. Also, for a detailed training program please see online Additional file 1.

#### “Vegeterranean” diet (V-med diet)

Briefly, the Med-diet is rich in olive oil, legumes, fish, chicken, nuts, milk products, fruits & vegetable. The modified V-Med diet in the study will be considered as ad-libitum (using fat sources) aimed for a minimum of 1 g/kg/day of protein, limiting carbohydrates: 3 servings for men and 2 servings for women per main meal and 1 serving per intermediate meal for both genders (medium/low glycemic index carbohydrates will be recommended). Subjects will be encouraged to consume ≥25 g of dietary fibers [37]. Subjects will be asked to avoid red meat and poultry completely and the consumption of dairy and eggs products will be limited to 2 servings per day. Three servings of fish should be consumed during the week. Legumes will comprise the source of the residual protein consumption, by the study’s team recommendations. The target legume consumption will be 0.75–1.5 cup per day (1–2 servings per day) of cooked beans, chickpeas or lentils and/or using pulses-based flour or pulses-based bread. Other sources for fibers will be recommended by consumption of whole grain carbohydrate foods (preferably millet, groats, bulgur, red-rice or “pseudo grains” like quinoa, buckwheat). Participants will be provided with a checklist of 15-g carbohydrate portions and of 10-g protein portions of recommended foods and the quantities they were expected to consume daily (as previously shown in [38]).

#### Empagliflozin (SGLT-2 inhibitor)

Subjects allocated to empagliflozin will be asked to add empagliflozin 10 mg, preferably in the morning, to their medication regimen. Subjects will be monitored for adverse events including hypoglycemic events during all study visits.

#### Measures of compliance/adherence

Compliance would be monitored in all three arms. Subject’s adherence to the exercise regimen will be assessed based on subject reporting and documentation in a dedicated booklet. Good compliance/adherence would be considered as performing ≥2/3 of original workout plan. Adherence to the V-Med diet will be based on a questionnaire scoring the diet implementation [39]. A score < 9 would be considered as low adherence, and ≥ 9 as high adherence. Adherence to the diet will also be assessed from 3-day food records or at least from 24 h recall report at the weeks 10 and 20 (visits 6, 9, respectively). For the drug arm, a recall of unused tablets would be performed after 10 weeks and at the end of the study. Consumption of less than 80% of study medication would be considered poor adherence to therapy.

### Follow up visits

Subjects will attend the IEMH at TASMC at the following intervals: screening Visit 1, Visit 2- Day 1- baseline day (allocation will be exposed at the end of baseline day; subjects allocated to SGLT-2 arm will meet the physician (RE) for instructions on the drug), Visit 3 Week 0- familiarization of protocol – meeting with the dietitian (AB) for V-Med arm only. Subjects allocated to the CRT arm will meet the physiologist (OK) or a physical instructor (AR) at their homes for familiarization. From familiarization day the 20-week count of the study period will start, and subjects will have an additional five “on site visits” at Week 1 (V-Med arm only; CRT – another visit at home after 1 week from beginning), Week 6 (V-Med arm only), Week 10 (all arms; CRT arm – end of study), Week 16 (V-Med arm only) and Week 20 (SGLTT-2 and V-Med arms) (Table 1).

**Table 1:** Study flow chart

	Screening	Baseline day (W0–1)^e^	Familiarization of protocol^b^ (W0–2)	After 1 week (W1)	After 6 weeks (W6)	After 10 weeks^c^ (W10)	After 16 weeks (W16)	After 20 weeks (W20)
On site visit number	1	2	3	4	5	6	7	8
Informed consent	X							
Eligibility criteria form	X							
Medical discharge and list of drugs from primary physician + physical activity confirmation letter	X							
Blood examinations from last 3 months (for eligibility)	X							
Physical examination for vital signs by study physician	X					X		X
Screening blood and urine tests (biochemistry and endocrinology): a) Urine microalbumin, sodium, creatinine, free cortisol) b) TSH, FT4 c) 25 OH vitamin D	X							
Demographic details, questions on health status, lifestyle using MABAT questionnaire	X							
Functional and physical activity questionnaires	X							
Food frequency questionnaire	X							
Guidance about diabetes diary	X							
Drug guidance for drug arm only		X						
Dietary guidance for diet arm only			X	X	X		X	
CRT guidance for CRT arm only			X	X				
CRT guidance for drug and diet arms						X		
Height		X						
Body weight		X				X		X
Body circumferences		X				X		X
Body fat %, lean and muscle mass assessments using BIA method		X				X		X
Resting blood pressure		X				X		X
Resting energy expenditure		X				X		X
24 h blood pressure monitoring		X				X		X
Fasting plasma glucose and HbA1C		X				X		X
Testosterone (T) and bioavailable T		X				X		X
HOMA- Insulin X glucose/23.5		X				X		X
LH, FSH, Estradiol		X				X		X
Diabetes diary (self-monitoring glucose levels, change in drugs)		X				X		X
Functional and frailty tests	X					X		X
CRT diary (report on training days and performance)			X	X	X	X	X	X
Left of tablets count of drugs						X		X
Adherence to MedD	X					X		X
Food diary – 3 days			X			X		X
Quality of life	X					X		X
Phone calls (adverse events monitoring)			X	X	X	X	X	X
Encouragement using phone calls			X	X	X	X	X	X

^a^Allocation concealment until the end of baseline day – then allocation is introduced both to researcher and the subjects. Subjects allocated to SGLT-2 would meet physician at the end of the day for instruction of the use of the drug and will be supplied with drugs for 20 weeks). Subjects allocated to Diet and CRT groups will make an appointment for the following week (familiarization)

^b^For diet subjects – on site visit and for CRT subjects – familiarization at their homes

^c^At this week CRT group will finish the study and SGLTT-2 and V-Med add CRT on top of their current intervention. Familiarization of the CRT will be performed on the same week.

*Abbreviations*: *BIA* Bioelectrical Impedance Analysis, *BMI* Body Mass Index, *CRT* Circuit Resistance Training, *HbA1c* Hemoglobin A1C, *MedD* Mediterranean Diet

### Main ascertainment of response variables

#### Anthropometric and sarcopenia

Anthropometric measurements will be obtained following a uniform protocol. Height, weight, waist and hip circumference will be measured twice. LBM, skeletal muscle mass (SMM), fat mass (FM) and % fat will be evaluated by the direct segmental multi-frequency bioelectrical impendence analysis (BIA) technique method using the ‘In-Body 770 body composition analyzer’. The In-Body 770 is a valid tool for the assessments of total body and segmental body composition [40]. Sarcopenia will be assessed in line with the consensus definition offered elsewhere [41] using three parameters: a) low skeletal muscle mass; b) low muscle strength; c) low physical performance [41]. Pre-sarcopenia will be defined as low skeletal muscle mass but normal strength and physical performance, whereas sarcopenia will be defined as having both low muscle mass with either low muscle strength or low physical performance. Severe sarcopenia will be determined by the presence of all 3 criteria (low muscle mass + strength + low performance). Skeletal muscle mass will be obtained by the In-Body (770) body composition analyzer. Shortly, the skeletal muscle index (SMI = skeletal muscle mass/body mass × 100) will be compared to gender-specific reference norms of young adults (aged 18–39 years). Sarcopenia will be defined as any value < 10.76 kg/m^2^ for men and < 6.76 kg/m^2^ for women [42]. Low muscle strength and low physical performance will be assessed using grip strength and gait speed test as described in the following paragraph on frailty.

#### Frailty

Will be assessed using a modification of the Fried assessment [43] based on low hand grip strength, low four meters average walking speed, low caloric expenditure on physical activity, self-reporting of extreme fatigue or low functionality and a spontaneous reduction of at least 4.5 kg in the past year. In this method, the low hand grip criterion is met when grip strength, assessed as the maximal result of 3 readings of the dominant hand by a handheld dynamometer [Jamar® Plus+ Digital Hand Dynamometer (Jamar® Smart) 200-lb.] is less than or equal to the gender- and body mass index–specific cutoff points provided by Fried et al. [43]. The slow gait speed criterion is met if the participant scored more than 4 s [44]. The 5-item FRAIL scale [45] which included the following 5 components: fatigue, resistance, ambulation, co-morbidity, and loss of weight. In both tools, frailty scores range from 0 to 5 (ie, 1 point for each component; 0 = best score, 5 = worst score) and are further categorized into: frail (3–5), pre-frail (1–2), and robust (0).

#### Functional tests

Other tests for functional ability using standardized protocols will be performed: time up and go test (TUG) [46], 30 s sit to stand (STS 30) [46], 30 s one leg stand [47], 2 min walk test [48], and isometric knee extension strength [49].

#### Glycemic control

FPG and HbA1c will be measured to evaluate metabolic control. At screening, Week 10 and Week 20 subjects will be asked to provide self-measured plasma glucose (SMPG) using blood glucose meters (see Table 1). SMPG is a measure of 7-time points: before breakfast; 120 min after start of breakfast; before lunch; 120 min after start of lunch; before dinner; 120 min after start of dinner; and at bedtime.

#### Ambulatory blood pressure monitoring

Twenty-four–hour ambulatory blood pressure (BP) monitoring (ABPM) will be measured with a fully automatic device (SpaceLabs 90207; Spacelabs Healthcare, Snoqualmie, WA). Daytime recordings are obtained every 20 min, whereas nighttime readings are collected every 30 min. BP variability (BPV) will be assessed as described elsewhere [50].

Sitting BP (diastolic and systolic) and pulse measurements will be preceded by at least 5 min of rest for the subject in a quiet setting without distractions (e.g. cell phones) and will be assessed in a sitting position with a completely automated device.

#### Nutritional intake

Based on the food records obtained by a registered nutritionist (AB) the total energy intake and the proportion of carbohydrates, proteins, and fats as well as several micronutrients will be calculated in the three interventional groups.

#### Quality of life

Will be self-reported and assessed by the SF-12 (a shorter version of the SF-36). The SF-12 yields two summary scores: physical component summary and mental component summary. The SF-12 is strongly correlated with the SF-36 [51]. The SF-12 have been translated and validated in Hebrew in inclusive studies of population aged 18–75 years [52].

#### Blood tests

Hormonal profile of each subject will be obtained at each study period and will include: LH, FSH, estradiol (E2), bioavailable and free testosterone, insulin, IGF-1 and 25-hydroxy vitamin D. FPG and HbA1C levels will be obtained from HMO’s records (up to 3 months before the study and at week 10 and 20). For future examinations frozen plasma and blood samples from patients will be stored and kept at − 20 °C and − 80 °C respectively.

#### Adverse events

An adverse event (AE) is considered as any unexpected and untoward event or medical occurrence in a participant that is related or not necessarily related to the treatment allocated in the study. AEs will be monitored in all study arms. Decision on the relation of event and the protocol will be made and documented. AEs will be classified by their severity (mild/ moderate/ severe), their causality (relation to the treatment: probable/ possible/ unlikely) and their outcome (resolved/ resolving/ resolved with sequelae – a lasting effect of the AE/ fatal). Every 2 weeks the subjects will be contacted by telephone and conduct an interview that will be documented in an AE log.

### Discontinuation

Participants may withdraw consent for any reason at any time or be discontinued from the trial by the investigator if based upon his clinical judgment, continuation in the trial is deemed inappropriate. In addition, a subject may be discontinued by the investigator if enrollment into the trial is inappropriate, the trial plan is violated, or for administrative and/or other safety reasons.

### Statistical methods

#### Sample size

The sample size calculation is based on the assumption that the mean difference in weight after 10 weeks between the empagliflozin arm and the CRT arm will be − 3.2 Kg. We assumed that the weight will not change in the CRT only group (body composition may change) and, based on the literature, that empagliflozin treatment causes a reduction in weight of ≈ 3–3.5 Kg. For 80% power and significance level of 5% each arm should include 40 subjects (*n* = 120 total). Another calculation was based on ANOVA for the comparison of three groups. We assumed that body fat % at the end of study will be 37, 36 and 40% for the diet, drug and CRT arms, respectively, with a 6% SD in each group. These assumptions are based on unpublished data obtained in a pilot study conducted in the IEMH. Assuming an attrition of ~ 10% we intend to recruit 120 up to 140 participants. Power calculations were performed using the G*Power Statistical Power Analyses for Windows [53].

#### Definition of main outcomes

Relative lean mass change will be calculated as % lean mass (the proportion of lean mass out of weight) at the end of study minus % lean mass at baseline divided by lean mass % at baseline and multiplied by 100. Change in body weight (%) will be calculated as body weight at the end of 10 weeks (and 20 weeks) minus baseline body weight divided by body weight at baseline and multiplied by 100.

#### Analysis

##### Data confidentiality

This is an investigator initiated unblinded study. Personal medical data will be available to the investigators. During data analysis and for presentation and publication purposes data will be anonymized.

##### Data handling

Data will be entered by AB. Quality control will be performed routinely by three students (AB, OK, and AR) using double data entry and range checks for data values. Analysis will be performed by SPSS software (IBM SPSS Statistics for Windows, Version 24.0; IBM Corp.). *P* values will be compared to the value of *p* = 0.05 or less (taking into account multiple comparisons using the Benjamini-Hochberg [54]).

##### Pre-analysis

Before analysis is performed, the following will be carried out/recorded for each continuous variable:

1. Minimum and maximum values (as part of quality control)
2. Mean ± standard deviation (SD)
3. Normality test (distribution and Kolmogorov-Smirnov)
4. Number of subjects included with no missing data
5. For categorical variables a proportion will be calculated and presented with the number of patients.

##### Baseline comparisons

To test differences in continuous variables between the study groups the one-way analysis of variance (ANOVA) test will be performed (or Kruskal–Wallis one-way analysis of variance when normal distribution is rejected). For comparison of dichotomous or categorical variables the Chi square test will be performed.

The primary endpoints (% change in LBM and change in weight) will be analyzed using the analysis of covariance (ANCOVA) after 10 weeks and after 20 weeks (for diet and drug arms only) including several factors and covariates. Table 2 indicates the list of covariates and factors. Table 3 indicates the different outcomes and appropriate statistical method.

**Table 2:** List of potential covariates and factors (partial list)

Factor or covariate	Type	Defined as
Treatment	Factor	SGLT2 inhibitor, diet, CRT
GLP1 agonists	Factor	Yes, no
Baseline body weight/ BMI	Covariate	–
HbA1c (%)	Covariate	–
Caloric consumption (Kcal)	Covariate	–
Resting metabolic rate (Kcal)	Covariate	–
Body fat %	Covariate	–
Strength (kg)	Covariate	–
Frailty or sarcopenia status	Factor	Frail or sarcopenic, pre-frail or pre sarcopenic and robust

*Abbreviations*: *BMI* Body Mass Index, *CRT* Circuit Resistance Training, *GLP-1* Glucagon-Like Peptide-1, *HbA1c* Hemoglobin A1C

**Table 3:** The different outcomes and appropriate statistical method (partial list)

	Endpoint type	Statistical method
Primary outcome		
Change in body weight (%) from baseline to 10 weeks or to 20 weeks	Continuous	ANCOVA
Change in % lean body mass from baseline to 10 weeks or to 20 weeks	Continuous	ANCOVA
Proportion of participants having lost ≥2.5% at week 10 and 20	Binary	Logistic regression
Secondary outcomes		
Change in waist circumference from baseline to 10 weeks or to 20 weeks	Continuous	ANCOVA
Change in FPG and HB1Ac from baseline to 10 weeks or to 20 weeks	Continuous	ANCOVA
Change in sarcopenia and frailty status (improved vs. not improved; change from one level to the other) at week 10 and 20	Binary	Logistic regression
Change in strength and other functional abilities from baseline to 10 weeks or to 20 weeks	Continuous	ANCOVA
Change in systolic blood pressure from baseline to 10 weeks or to 20 weeks	Continuous	ANCOVA
Change in quality of life (improved vs. not improved; change from one level to the other) at week 10 and 20	Binary	Logistic regression

*Abbreviations*: *ANCOVA* Analysis of Covariance, *FPG* Fasting Plasma Glucose, *HbA1c* Hemoglobin A1C

Primary analysis will be intent to treat analysis. It will include all patients that had been randomized, started protocol and reached the end of the trial examinations (or last observation available will be imputed as their “end of study value”). Per protocol analysis will be performed by the a-priori conditions: a) for participants who had high compliance to workout (≥ 2/3 of sessions); b) for participants who had high compliance to diet (≥ score 9 in the Med-diet score and/or caloric consumption change from baseline was not more than 15% of report at baseline); c) for participants who had high compliance to drug therapy (≥80% tablets taken); d) for participants who did not violate the protocol (for example: changed thier habitual diet in the CRT arm; initiated new physical activity other than the one prescrived by us, ect.).

Sensitivity analysis can include all the participants who finished the trial including dropouts, with imputation of the last observation. This will be compared to an analysis which includes only participants that fully completed the study (have an assessment at the end of study).

We may also perform a mixed model for repeated measurements based on assessments of participants who completed the study and had high compliance to the treatment. An unstructured covariance matrix for measurements within the same participant will be employed, assuming that measurements for different participants are independent. The covariates and factors in Table 2 will be nested in the model.

Last, subgroup analysis will be done by stratification to several factors shown in Table 2, where ANCOVA or logistic regression models will be performed according to the outcome assessed (Table 3).

## Discussion

Our study aims to evaluate the effectiveness of three different interventions (V-Med diet, CRT and empagliflozin, separately or in combination) on metabolic, anthropometric and physical function parameters in older subjects with T2DM. Several new facets of T2DM treatment in this age group are at the core of this study: 1. This is the first comparison of empagliflozin to lifestyle modification in older patients; 2. This is the first evaluation of a multistage lifestyle and empagliflozin therapy interventions on frailty and sarcopenia; 3. Our hybrid diet intervention (V-Med diet) has thus far not been tested in conjunction to CRT or compared to other treatment modalities; 4.This is the first study to assess the effect of a CRT program in older subjects with T2DM.

There are, however, several limitations to our study protocol: 1) the possibility for volunteer bias which might lead to the inclusion of highly engaged patients. This may affect only the external validity of the study, but not its internal validity; 2) the exclusion criteria include several co-morbidities that are likely prevalent in the older age and associated with diabetes such as recent stroke or myocardial infraction. While this may minimize the generalization of our results, subjects with these conditions may not benefit from our interventions; 3) the apparently narrow HbA1C range used as an inclusion criterion (6.5–8%), may also limit the generalization of our results at this age group. Still, we wish to minimize the effect of major reduction in glycemic values per se (associated with higher values of HbA1C at entry for the study) as a potential contributor to any observed improvements in functional and metabolic states; 4) given its relatively short duration and limited sample size the study is unlikely to evaluate morbidity outcomes such as the incidence of myocardial infraction or stroke.

The combination and comparison of the three interventions for metabolic control may lead to better understanding of their mechanism of action with potential clinical implications in older subjects with T2DM. This study may also provide evidence of the effectiveness of different interventions on the progression from diabetes to sarcopenia and/or frailty and their potential effect on the quality of life older subjects with T2DM.

## Additional file

Additional file 1: Circuit resistance training protocol. (PDF 3533 kb)

## Data Availability

Not applicable.

## Abbreviations

30-STS: 30 s Sit To Stand
ABPM: Ambulatory Blood Pressure Monitoring
AE: Adverse event
ANCOVA: Analysis of Covariance
ANOVA: One-way analysis of variance
BIA: Bioelectrical Impedance Analysis
BMI: Body Mass Index
BP: Blood Pressure
CRT: Circuit Resistance Training
DEXA: Dual Energy X-ray Absorptiometry
FM: Fat Mass
FPG: Fasting Plasma Glucose
GLP-1: Glucagon-Like Peptide-1
HbA1c: Hemoglobin A1C
IEMH: Institute of Endocrinology Metabolism and Hypertension
LBM: Lean Body Mass
MedD: Mediterranean Diet
PA: Physical Activity
RCT: Randomized Controlled Trial
RMR: Resting Metabolic Rate
SD: Standard Deviation
SGLT-2: Sodium-Glucose Transporter-2
SMI: Skeletal Muscle Index
SMM: Skeletal Muscle Mass
SMPG: Self-Measured Plasma Glucose
TASMC: Tel Aviv-Sourasky Medical Center
TUG: Time Up and Go test
T2DM: Type 2 Diabetes Mellitus
V-Med diet: Vegeterranean Diet
