# Supplementary material for: The effect of circuit resistance training, empagliflozin or “vegeterranean diet” on physical and metabolic function in older subjects with type 2 diabetes: a study protocol for a randomized control trial (CEV-65 trial)
Source: BMC Geriatr. 2019 Aug 22;19:228. doi: 10.1186/s12877-019-1219-7 (PMC6704502; doi:10.1186/s12877-019-1219-7)
Supplement: Supplementary file 1 — Circuit resistance training protocol. (PDF 3533 kb) [file 12877_2019_1219_MOESM1_ESM.pdf]

Circuit resistance training protocol

General

- To maintain a positive training stimulus to maximize exercise outcome, the CRT program is progressive in nature. To accomplish that, the relative exercise intensity will be maintained through periodic increases in exercise volume (# of cycles) or intensity (RPE) in accordance with the program's phases.
- In addition, the subject will be encouraged to gradually increase the depth (range of motion) of the body weight exercises performed for the lower body as he/she will become more flexible, confident and efficient in performing the exercises.

Visits

Initial visit

- Only two home visits will be done by the researcher throughout the CRT program to, emphasize the feasibility of accomplishing a truly minimally supervised program.
- An initial home visit will be held within one week after completion of the subject's final visit to the department.
- The researcher will explain and demonstrate to the subject the correct form to perform all the exercises prescribed in the program as well as the structure of the exercise session including the warm up, resistance exercises and stretching exercises.
- The researcher will teach the subject how to gauge exercise intensity using the BORG RPE scale, corresponding to the intensity prescribed in the exercise program
- An elastic resistance band corresponding to the correct RPE prescribed in the program will be chosen by the researcher after performing a few repetitions in each exercise by the subject and observing his level of effort while performing the exercises. The subject will be given instructions to change to a tougher resistance band color once exercise intensity falls below that specified in the program.
- Specific instructions will be given to the subject to engage core musculature and correct footing to maintain postural control and proper elastic band tension.
- The researcher will, show the subject the correct position to place the resistance band door adaptor on the outside face of the subjects main home door, to perform the resistance bands exercises correctly and insure correct implementation by the subject.
- The researcher will observe a complete exercise session performed by the subject and provide immediate feedback as necessary.
- At the end of the exercise session the researcher will handout the subject an exercise log where information regarding session completion will be filled by the subject and insure correct implementation. Also, the researcher will instruct the subject to fill the exercise log immediately after he has completed an exercise session.
- The researcher will provide appropriate breathing instruction to be performed by the subject during the exercises, specifically: inhaling before a lift, exhaling as each repetition is commenced (concentric phase) and inhaling while returning to each repetition starting position(eccentric phase).
- An additional visit by the researcher with the subject will be scheduled at that time, that will be performed within a week from the initial visit.
- The researcher will give the subject his personal communication information in case questions arise while performing the program. Also, the researcher will inform the subject of a biweekly phone call that will be initiated by the researcher and its potential content.

Second visit

- A second and final visit will be held approximately one week after the first.
- the researcher will check if the subject filled the exercise log correctly and provide appropriate feedback as necessary.
- The researcher will observe the subject perform a complete exercise session and provide appropriate feedback as necessary.
- The researcher will show the subject the program's progression including the addition of another cycle at week 5 and increasing intensity by week 10.

Biweekly phone call

- A biweekly phone call will be initiated by the researcher to the subject to insure high motivation is maintained by the subject and that the correct exercise intensity is withheld according to the program's phase.
- The researcher will address any technical and administrative issues encountered by the subject.
- The researcher will confirm that the subject is filling the exercise log as instructed.
- The research will call the subject at the correct time in order to confirm the subject has increased exercise volume or intensity, according to the program's phases.

Exercise log filling

The subject will fill the following information in the exercise log:

- Date and time of completed session
- Color of elastic resistance band used
- RPE of exercises
- Number of circuits
- General filling
- Additional remarks including pain, physical discomfort or any other relevant information

The subject will receive the following items:

1. A booklet with the exercise program (figure 2') along with the prescribed exercise illustrations and exercise instructions (appendix 1')
2. An individually prescribed elastic rubber band corresponding to the subject's current strength level along with an additional elastic band with added resistance (color)
3. A door adopter to connect resistance band safely to door
4. An exercise log to record individual exercise sessions completed including remarks on session RPE

Safety measures

1. The subject will be advised to perform the exercise session wearing walking/jogging shoes, while wearing comfortable and cool clothing.
2. The subject will be advised to make sure the exercise area is free of potential obstacles, and the room is cool and sufficiently aired.
3. The subject will be advised not to exercise on a full stomach. While on the side not to be than 5 hours post.
4. The subject will be advised to exercise at a time of his convenience, preferably at the same time of day, yet not to close to waking up or sleep time.
5. Subjects with painful conditions will be advised to exercise only in a pain-free range of motion and contact the researcher if a new pain is observed in previously pain free area or range of motion.
6. The subject will be advised that in the case the following sensations occur during or after exercise, he will report immediately to a medical facility: dizziness, chest pain, extreme fatigue, shortness of breath. After his discharge the subject must contact the researcher, inform him of the medical findings and discuss the required course of action.
7. In case of an unusual mild pain occurring during a specific exercise, the subject will continue with other exercises that do not cause pain and contact the researcher later for further instructions.
8. The subject will be advised that in the case he/she feels unwell due to a temporary illness such as a cold or flu, training must be postponed until exercise can be resumed. In a case illness has interrupted the continuity of the Programme for more than 4 days, the subject should contact the researcher before resuming the program.
9. The subject will be advised that he should continue to take his regular medications as scheduled.
10. If the subject has incurred a serious fall followed by a strong pain sensation or disability, he should see his doctor and notify the researcher. If the subject is cleared to resume the program, the researcher might provide additional instruction by phone.

11. Due to the possibility that performing the CRT program might cause a hypoglycemic event, the subject should prepare beforehand some sugar and a measuring kit for quick response. Any change in awareness or an out of focus sensation might indicate a low sugar level. In that case he/she must stop exercising, check his/her blood sugar level and consume sugar as taught by their personal doctor.
12. It is recommended that for subjects needing added support while performing the standing exercises, grasping a chair will be used for added safety and support.

General program layout according to phases

Table 1. Frequency: *3 weekly exercise sessions performed on nonconsecutive days*

Phase	Timeline (Weeks)	Intensity (RPE)	Volume (N of cycles)	Exercises (N)	Repetitions (N)	Tempo (sec')	Rest between Exercises and cycles (sec')	Duration (min')
1	1-4	13-14	2	9	10	2-0-2	Short (≤15)	~ 20
2	5-8	15	3	9	10	2-0-2	Short (≤15)	~ 25.5
3	9-10	15	3	9	15	2-0-2	Short (≤15)	~ 32

- Session structure: warm up exercises followed by the prescribed # of cycles (corresponding to the program's phase) culminating in cooldown/stretching.
- Phase 1 (1-4 weeks): Two cycles 10 repetitions/exercise. Total session time: warm up (4.5 M') + cycle 1 (5.5 M') + cycle 2 (5.5 M') + stretching (3.5M'). total session time ~ 20 minutes
- Program phase 2 (5-8 weeks): Three cycles 10 repetitions/exercise. Total session time: warm up (4.5 M') + cycle 1 (5.5 M') + cycle 2 (5.5 m') + cycle 3(5.5 m') + stretching (3.m'). Total session time ~ 25.5 minutes
- Program phase 3 (9-10 weeks): Three cycles 15 repetitions/exercise. Total session time: warm up (4.5 M') + cycle 1 (8 M') + cycle 2 (8 M') + cycle 3 (8 M') + stretching (3.5M'). Total session time ~ 32 minutes

Table 2. *Exercise program*

Exercise		Repetitions	ERB	Intensity/pace	Duration (Sec)	Remarks
Stepping in place	▶1	20 Steps / each leg		RPE 10	30	Comfortable pace, Keep balance
Side to side stepping	▶2	15 Steps / each side		RPE 12	30	Comfortable pace, Keep balance
backward arm circles	▶3	10 rotations / each side		Own pace	30	Full range circles
Stepping in place		20 steps / each side		RPE 12	30	Comfortable pace, Keep balance
Side to side stepping		15 Steps / each side		RPE 12	30	Comfortable pace, Keep balance
backward arm circles		10 rotations / each side		Own pace	30	Full range circles
Hip rotations	▶4	7 rotations /each side		Slow	20	Comfortable pace
Side to side static lunge	▶5	7 transfers /each side		Slow	20	Comfortable range and pace
Dynamic side bend	▶6	7 repetitions / each side		Own pace	20	Comfortable range and pace

RESISTANCE TRAINING CYCLE

Exercise		*Repetitions*	*ERB*	*Pace (sec)*	*Duration (sec)*	*Remarks*
Chair Sit to stand	▶7	*10*		*4*	*40*	*Feet shoulder width*
Standing row	▶8	*10*	*ERB*	*4*	*40*	*Straight back. One leg forward*
Standing shoulder EXT	▶9	*10*	*ERB*	*4*	*40*	*Keep balance*
Lunge to one side	▶10	*10*		*4*	*40*	*Hold chair. Straight back*
Lunge to other side		*10*		*4*	*40*	*Hold chair. Straight back*
Standing chest press	▶11	*10*	*ERB*	*4*	*40*	*Straight back. One leg forward*
Standing reverse fly's	▶12	*10*	*ERB*	*4*	*40*	*Feet shoulder width. Arms straight*
Standing wide row	▶13	*10*	*ERB*	*4*	*40*	*Straight back. One leg forward*
Standing biceps curl	▶14	*10*	*ERB*	*4*	*40*	*Straight back. One leg forward*
Single Leg Toe Raises	▶15	*10 each side*		*4*	*80*	*Reach maximum height*
Side to side stepping		*15 Steps / each side*			*30*	*Comfortable pace, Keep balance*

***Phase 1'** - (week 1-4) Perform 2 resistance cycles continuously*

***Phase 2'** - (week 5-8) Perform 3 resistance cycles continuously*

***Phase3'** - (week 9-10) Increase number of repetitions to 15 in all RT exercises*

***STRETCHING** - (should be performed at the culmination of the last cycle corresponding to the program's phase*

Backward arm circles		*10 rotations / side*	*Own pace*	*30*	*Full circle*
Dynamic side bend		*Each side*	*20 seconds*	*40*	*Pleasant stretch*
Seated quadriceps stretch	▶16	*Each side*	*20 seconds*	*40*	*Pleasant stretch*
standing hamstring stretch	▶17	*Each side*	*20 seconds*	*40*	*Pleasant stretch*
Seated chest stretch	▶18	*Each side*	*20 seconds*	*20*	*Pleasant stretch*
Standing calf stretch	▶19	*Each side*	*20 seconds*	*40*	*Pleasant stretch*

Borg's rate of perceived exertion (RPE) scale

Borg G. Perceived exertion as an indicator of somatic stress. Scand J Rehabil Med. 1970;2(2):92-8

1

Walking in place

Walk in place with arm bent at 90 degrees

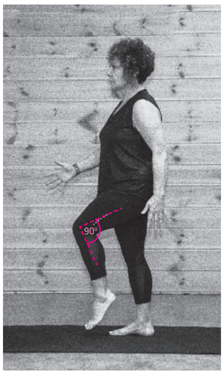

2

Steping side to side

With arms held at the waist take a step from side to side keeping your balance

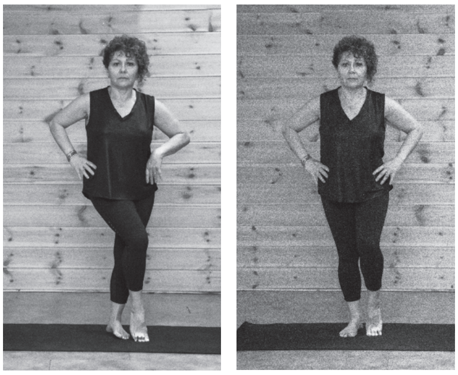

3

Backwards arm circles

While standing stable move arms in a full circular motion

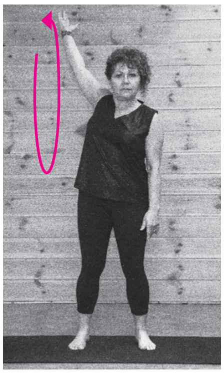

4

Hip rotations

standing stable with legs shoulder width apart rotate hips in a circular motion from left to right and then from left to right

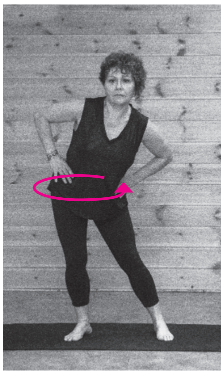

5

Dynamic lateral knee bend

stand in a wide lateral stance, shift body weight from side to side by bending one knee and straightening the other. Make sure knee follows foot position

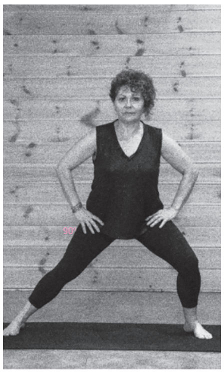

6

Dynamic Side flexion

Standing stable with legs shoulder width apart, extend one arm overhead and while bending at the waist pulsate lightly to the other side keeping hips static

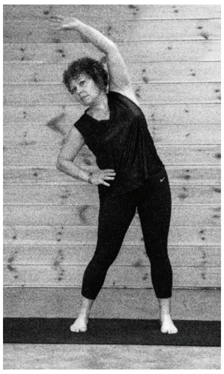

7

Chair Squat

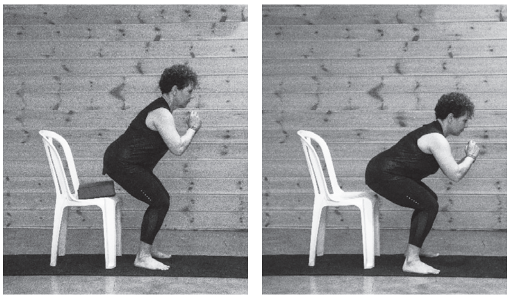

Stand behind a chair with feet should width. Start sitting down while keeping back straight and knees over feet. Just before fully sitting down stand up again. To reduce stress on knees its possible to use a higher chair or add some height to the chair

8

Standing narrow grip row

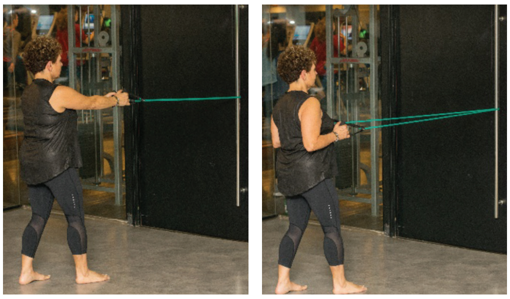

Facing door with one foot forward and extended arms, pull band towards you stiffening your body with elbows facing the floor till they are bent to 90 degrees, Return to starting position

9 Standing shoulder extension

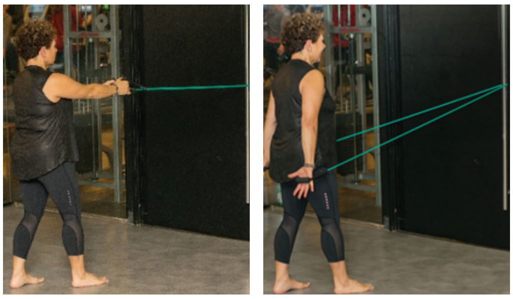

Facing door with one foot forward and extended arms, pull band toward you at shoulder height, till elbows bent to 90 degrees, stiffening your body. Return to starting position

10 Supported in place lunge

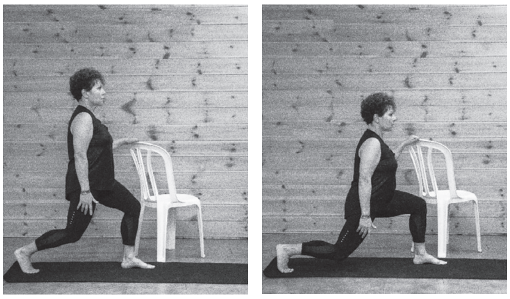

Standing next to a chair, take a long stride holding the chair for support. Keep the back straight and bend both knees lowering yourself almost till knee touches floor. Rise to standing position

11 Standing chest press

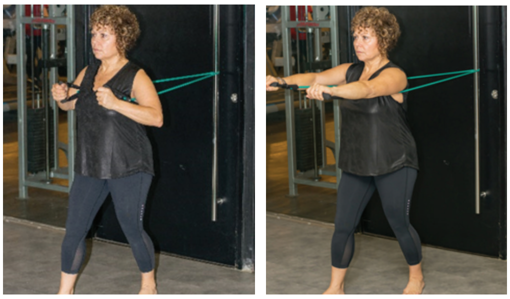

Stable with one foot forward, back to the door and elastic band at chest level, extend the elbows while stiffening the body and return to starting position

12 Standing reverse flys

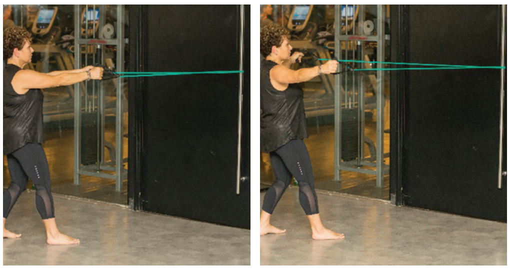

Facing door with one-foot forward arms extended, pull band backwards with straight arms you at shoulder height, till arms are at your sides, while stiffening your body. Return to starting position

13 Standing wide grip row

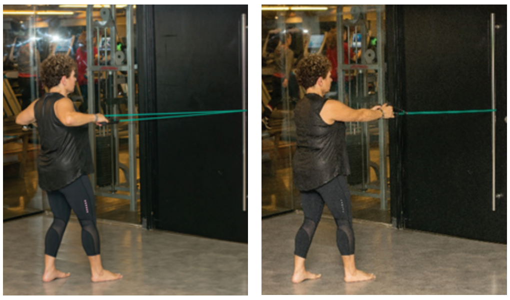

Facing door with one-foot forward arms extended, pull band toward you at shoulder height, till elbows bent to 90 degrees, stiffening your body. Return to starting position

14 Standing biceps curl

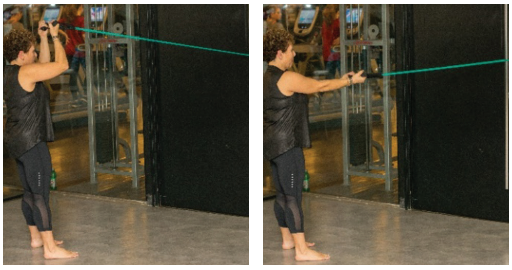

Facing door stable with feet shoulder width forward and extended arms, pull band toward you at shoulder height, keeping elbows high, till elbows bent to 90 degrees, stiffening your body. Return to starting position

15 ▶ **Standing one leg heel raise**

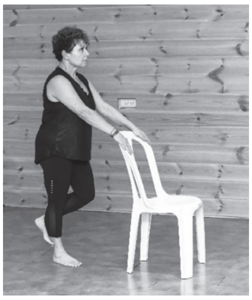

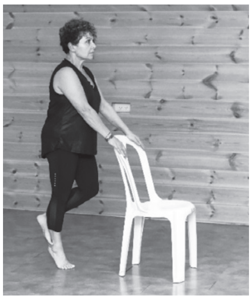

Standing behind a chair. Hold chair in both hands and while standing balanced on one foot raise yourself on your toes to the highest point possible keeping knees straight. Return to starting position

16 ▶

Seated quadriceps stretch

While sitting sideways on the edge of a chair, grasp outside leg with outside arm, and bring heel toward buttocks till reaching a pleasant stretch. Hold position and breath

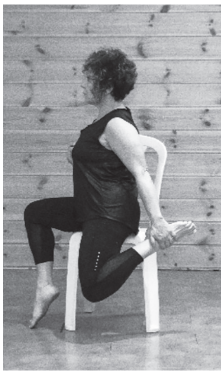

17 ▶

Standing hamstring stretch

While standing using a chair for support, bring one leg forward and try to extend the knee till you feel a comfortable stretch. Hold position and breath

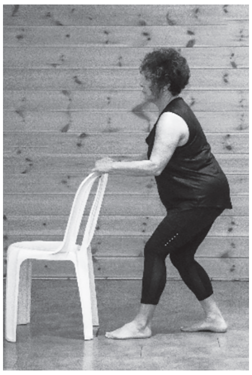

18 ▶

Seated chest stretch

While sitting with a straight back leaning against the back of a chair, put both hands behind ears, squeeze shoulder blades together while opening your chest, feeling a comfortable stretch. Hold position and breath.

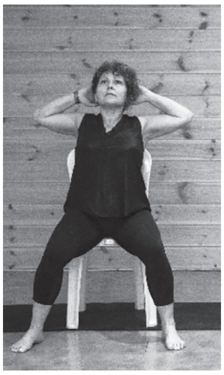

19 ▶

Standing calf stretch

While supported by the back of a chair, extend one leg backwards, heel on the flor and knee straight. Bend forward leg at the knee till you feel a comfortable stretch. Hold position and breath.

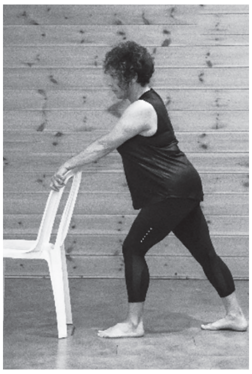

Week #	Session #	Date	Time	Rpe	Band Collor	Cycles	Remarks
Week 1	1						
	2						
	3						
Week 2	4						
	5						
	6						
Week 3	7						
	8						
	9						
Week 4	10						
	11						
	12						
Week 5	13						
	14						
	15						
Week 6	16						
	17						
	18						
Week 7	19						
	20						
	21						
Week 8	22						
	23						
	24						
Week 9	25						
	26						
	27						
Week 10	28						
	29						
	30						

Borg's Rate of perceived exertion scale

Rating	***Perceived exertion***
6	*No exertion*
7	*Extremely light*
8	
9	*Very light*
10	
11	*Light*
12	
13	*Somewhat hard*
14	
15	*Hard*
16	
17	*Very hard*
18	
19	*Extremely hard*
20	*Maximal exertion*

Borg G. Perceived exertion as an indicator of somatic stress. Scand J Rehabil Med. 1970;2(2):92-8
